# Guided Adaptive Diffusion: An Evolutionary Framework for Multimodal Atomistic Structure Prediction

**DOI:** 10.1021/acs.jcim.6c00843

**Published:** 2026-06-25

**Authors:** Alexander Adel, Jakub Szmitek, Benedikt Hartl, Ralf Wanzenböck, Georg K. H. Madsen

**Affiliations:** † Institute of Materials Chemistry, 9142 TU Wien, Vienna 1060, Austria; ‡ 27259 Allen Discovery Center at Tufts University, Medford, Massachusetts 02155, United States; § Institute of Theoretical Physics, TU Wien, Vienna 1040, Austria

## Abstract

Atomistic structure prediction requires search algorithms capable of locating global and local minima on high-dimensional, multimodal potential energy surfaces. Traditional algorithms tend to become less effective as the dimensionality of the search space increases. In this work, we introduce an adaptive diffusion framework that reinterprets the neural-network-based denoising process as an evolutionary search mechanism for structure optimization. The framework incorporates two key optimization mechanisms. First, geometric constraints provide physics-informed guidance during sampling. Second, a memetic approach combines the global diverse sampling capabilities of diffusion models with local gradient-based relaxation. Unlike heuristic evolutionary algorithms, which rely on predefined analytical update rules for comparatively simple search distributions, neural-network-based denoising learns the underlying structure of the search space directly from the full accumulated history of sampled configurations, enabling the representation of highly complex distributions. We benchmark the algorithm using Lennard-Jones and gold clusters, demonstrating its ability to locate the global minimum and an ensemble of low-energy local minima within a single evolutionary run. The results indicate that the algorithm remains effective on high-dimensional potential energy surfaces, maintaining both population diversity and search efficiency throughout the optimization.

## Graphical abstract

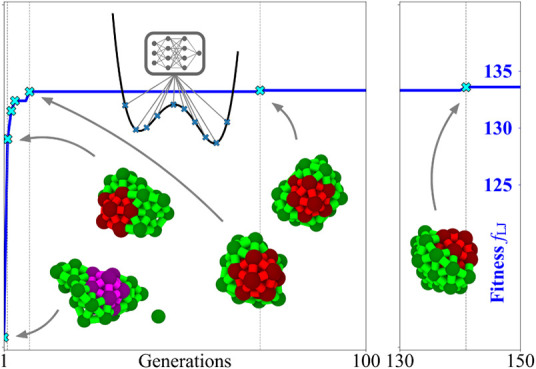

## Introduction

Atomistic structure prediction is essential for the design and discovery of novel advanced materials.^1–3^ Powerful exploratory computer algorithms are necessary to find diverse sets of stable minima on the often high-dimensional and multimodal potential energy surfaces (PES).

Evolutionary algorithms are heuristic methods capable of efficiently navigating rugged problem spaces for global optima via evolution-inspired selection and replication mechanisms acting on populations of candidate solutions.^4^ They have proven highly effective for structure optimization tasks.^5–7^ A well-established example is the Covariance Matrix Adaptation Evolution Strategy (CMA-ES),^8,9^ a stochastic method for the optimization of real-valued, nonlinear, and nonconvex functions. Sets of candidate solutions are sampled from a multivariate normal distribution and are evaluated using an objective function, e.g., a PES for atomistic configurations. Generation by generation, the covariance matrix is adapted to increase the probability of sampling high-fitness solutions, effectively steering the distribution toward regions of stable configurations. CMA-ES has been successfully applied to materials problems such as point defects^10,11^ and surface reconstructions.^12,13^

While algorithms such as the CMA-ES can converge quickly, their effectiveness becomes increasingly limited in high-dimensional optimization tasks.^14^ Attempts to mitigate this limitation include learned self-adaptation schemes, in which algorithmic parameters are dynamically adjusted based on information extracted from previous search trajectories.^15,16^ However, the underlying search distribution inherently restricts the ability to represent complex high-dimensional landscapes.

In contrast, generative machine-learning models have emerged as a promising technique for structure generation.^17,18^ In particular, diffusion probabilistic models^19–24^ have attracted considerable attention due to their relatively stable training and their ability to condition their output to desired properties.^25–27^ As flexible generative frameworks, diffusion models learn statistical structure directly from high-dimensional data and can approximate complex distributions. By iteratively denoising random initial configurations, diverse, high-quality samples can be generated without requiring explicit assumptions about the functional form of the underlying distribution. These capabilities have led to successful applications across a wide range of molecular systems^28–32^ as well as periodic crystalline materials.^33–39^

Current generative structure prediction approaches in material science typically follow standard machine-learning workflows, in which models are first trained on predefined data sets and subsequently used as black-box generators to sample new configurations that statistically conform to the underlying training data distribution. Most existing approaches thus rely on models pretrained on extensive databases of known structures and compositions. While effective in data-rich settings, such approaches are intrinsically biased toward regions of configuration space already well represented in the training data.

In contrast, we propose a lightweight search framework based on an adaptive diffusion strategy. Recently, the reverse diffusion, or denoising, process has been reinterpreted as an optimization procedure, in which sampling is guided toward high-fitness solutions rather than reproducing target data distributions.^40–42^ Our framework is based on a recently introduced algorithm for iteratively refining the reverse diffusion across successive generations^40^ and operates without pretraining on known structures.

There is both a mathematical and an intuitive correspondence between adaptive diffusion and evolutionary strategies.^40,41^ However, adaptive diffusion strategies offer more powerful representational flexibility. Instead of updating a single multivariate Gaussian, they employ neural networks to learn the structure of the search distribution directly from sampled configurations, enabling the representation of complex correlations and multiple basins. Furthermore, the learning-based adaptation leverages the full history of the sampled structures rather than relying on heuristic covariance updates, allowing diffusion models to maintain diverse representations while focusing sampling on high-fitness regions.

In the present work, we test the adaptive diffusion approach on Lennard-Jones and gold clusters and demonstrate how the approach identifies diverse sets of low-energy configurations, including global minima as well as a wide range of competing local minima. The Lennard-Jones (LJ_*n*_) clusters, with their large number of closely competing local minima, have been used as benchmarks for optimization algorithms for over five decades.^14,43–47^

The gold clusters (Au_*n*_) represent another group of extensively studied systems characterized by a remarkable diversity of stable configurations, namely the transition and noble metal nanoclusters.^48–50^ For neutral gold clusters, the nature of the lowest energy configurations depends intricately on size. They transition from shell-like flat-cage motifs to spherical-like hollow-cage structures at Au_17_ ^51^ followed by fcc-like pyramidal structures at Au_19_.^52^ Au_20_ is seen as a magic cluster,^53^ since it forms a highly stable tetrahedral structure.^54–57^ Beyond Au_20_ a rich competition between fcc-like pyramidal, shell-like flat-cage, spherical hollow-cage, tubular cigar-shaped, and stuffed or amorphous motifs can be observed,^58–65^ making gold clusters a stringent and chemically realistic test case for global optimization algorithms.

The paper is organized as follows. First, we introduce how adaptive diffusion models can be utilized for optimization problems and the extensions necessary to make the algorithm effective for structural optimizations. Next, results for the LJ clusters are presented as benchmarks that highlight the advantages of the new algorithm compared to evolutionary strategies. Finally, neutral gold clusters are investigated by the algorithm, using a machine-learned foundation model as the energy backend. The paper ends with a conclusion and an outlook.

## Background

### Diffusion Models

Conventionally, generative diffusion models apply a stochastic forward process that progressively transforms data into a tractable prior and learn the corresponding reverse process that removes the noise. In turn, the learned reverse process is used to iteratively refine noisy initial configurations into structured data with unmatched sample quality and diversity, positioning diffusion models as powerful generative engines for sampling from complex, high-dimensional distributions.^19–24^

The forward process is constructed as a Markov chain that progressively adds Gaussian noise ϵ∼N{0,I} according to a decreasing variance schedule α_*t*_ ∈ [0, 1] over a number of uniformly distributed discrete time steps *t* ∈ [0, ..., *T*]. This construction admits a closed-form expression for sampling the latent variable ***x***_*t*_ directly from the original data ***x***_0_

$$q\left(x_{t}|x_{0}\right)=N\left\{x_{t};\sqrt{A_{t}}x_{0},\left(1-A_{t}\right)I\right\}$$

with $A_{t}=\overset{t}{\underset{s=1}{\prod }}\alpha_{s}$ . Thereby, ***x***_*t*_ can be written as a linear combination of the original data and the added noise

1 $$x_{t}=\sqrt{A_{t}}x_{0}+\sqrt{1-A_{t}}\epsilon$$

The reverse process is modeled by a neural network ϵ_θ_(***x***_*t*_, *t*) with parameters θ, which is trained to predict the noise ϵ present in corrupted data ***x***_*t*_ at any given step *t*. In practice, the forward process in eq 1 is used to augment training data ***x***_*t*_ from data points ***x***_0_ ∼ *q*(***x***_0_). The network parameters are optimized by minimizing the loss function

2 $$L\left(\theta \right)=E_{x_{0},t,\epsilon }{∥\epsilon_{\theta }(x_{t},t)-\epsilon ∥}^{2}$$

with stochastic gradient descent.

This choice of the diffusion process allows the reverse process to be expressed as a sequence of conditional Gaussian transitions

$$p_{\theta }\left(x_{0:T}\right)=p\left(x_{T}\right)\overset{T}{\underset{t=1}{\prod }}N\left\{x_{t-1};\mu_{\theta }\left(x_{t},t\right),\Sigma_{\theta }\left(x_{t},t\right)\right\}$$

starting with complete noise $p\left(x_{T}\right)=N\left\{x_{T};0,I\right\}$ . Trained diffusion models approximate the training-data distribution *p*_θ_ ≈ *q*(**x**_0_) and thus permit sampling new instances ***x̂***_0_ ∼ *p*_θ_. Such samples are obtained by reversing the diffusion process: starting from pure noise ***x***_*T*_, corrupted data is iteratively refined ***x***_*t*_ → ***x***_*t*–1_ by applying

$$x_{t-1}=\frac{1}{\sqrt{\alpha_{t}}}\left(x_{t}-\frac{1-\alpha_{t}}{\sqrt{1-A_{t}}}\epsilon_{\theta }\left(x_{t},t\right)\right)+\sigma_{t}z$$

with $\sigma_{t}=\sqrt{1-\alpha_{t}}$ and z∼N{0,I} .

### Adaptive Diffusion Strategies

Hartl et al.^40^ introduce a strategy in which the reverse diffusion process is reinterpreted as a population-based optimization algorithm. In contrast to conventional diffusion models, no forward diffusion process tied to a fixed data distribution is performed. Instead, the denoising model itself is iteratively refined and used as a generative search distribution. The framework samples a population ***G***_τ_ = {***x***_τ,1_, ..., ***x***_τ,λ_} with λ individuals using a diffusion model $G_{\tau -1}$ . The diffusion model is then retrained using samples from a fitness-weighted subset of the entire collection of populations ***C***_τ_ = {***G***_1_, ..., ***G***_τ_}. This introduces the fitness *f* of the sampled individuals as a weighting factor in the loss *L*(θ) in eq 2,

3 $$L_{\mathrm{evo}}\left(\theta \right)=E_{x,t,\epsilon }h\left[f\left(x\right)\right]{∥\epsilon_{\theta }(x,t)-\epsilon ∥}^{2}$$

prioritizing high-fitness samples via the mapping *h*. Here, the potential energy –*E*_pot_ is used as the fitness measure of atomistic particle configurations ***x***, while the mapping

4 h[f(x)]=exp(βf(x))

corresponds to a weighting according to the Boltzmann distribution with inverse temperature β. In the present context, however, the β parameter should be mainly interpreted as an evolutionary pressure that controls the trade-off between selection and diversity.

The resulting algorithm represents an evolutionary strategy that sequentially refines a governing generative model across generations to sample better-adapted offspring via fitness-weighted guidance, while the stochastic diffusion process facilitates variation.^40,41^ It highlights intriguing parallels between biological evolution and learning.^66–69^ However, unlike a conventional evolutionary strategy like the CMA-ES, which iteratively adapts a single Gaussian distribution, the neural-network-based denoising allows diffusion models to represent highly complex multimodal distributions, see Figure 1. Furthermore, the guidance emerges from learned representations rather than heuristic rules for covariance adaptation.

**Figure 1:**
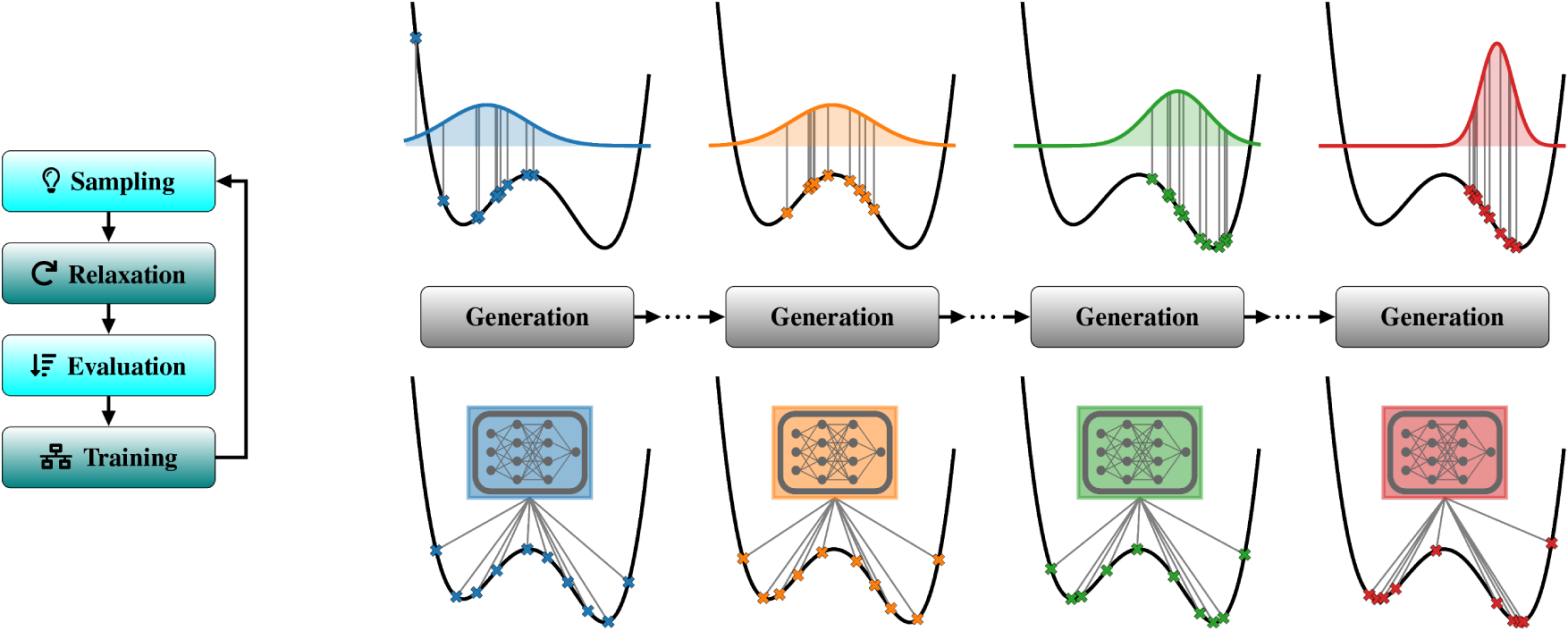
Comparison between the sampling processes of CMA-ES and the adaptive diffusion strategy. **(Left)** The diagram illustrates the steps performed by the adaptive diffusion strategy in every generation (see text for details). **(Top right)** CMA-ES uses a Gaussian distribution to sample new configurations, as can be seen in the schematic plots. This choice restricts the later generations (when the covariance matrix already reduced the distribution to a narrow area) to a single mode of the potential energy surface. **(Bottom right)** The diffusion model trains a new neural network in every generation, thereby increasing the flexibility of the sampling process. The schematic visualizations show that the model retains the ability to sample a number of different modes also in the later generations, while at the same time dedicating additional samples to the exploration of the PES.

### Algorithm

Here, we extend the adaptive diffusion strategy^40^ by two key mechanisms to improve its applicability to structure prediction.

First, vanilla diffusion models lack access to explicit physical or chemical information. Especially in a low-data regime, sampling can be fragile and easily yield unphysical configurations with very low fitness when solely trained on atomistic coordinates. We therefore either bias (i) the final atomistic positions via classifier-free conditioning by imposing spatial restrictions or (ii) positional updates during sampling via physics-informed gradient guidance. While classifier-free conditioning can be seen as a soft constraint that biases the diffusion model’s predictions toward solutions that exhibit desired features, gradient guidance applies physical forces during the sampling process to confine the solutions.

Second, although diffusion models excel at learning the broad distribution underlying the training data, they can lack precision when sampling fine details. Leveraging highly accurate and computationally efficient force fields available in atomistic simulations, we here propose (iii) a memetic approach^70–72^ —i.e., an evolutionary strategy augmented with local gradient-based refinement between sampling and evaluation—that combines the global (diverse) sampling capabilities of diffusion models with local gradient-based optimization, see left panel of Figure 1. By carefully relaxing sampled configurations, we not only increase the sample quality and thus the training data but also allow the diffusion model to focus on characteristics of the atomistic input data, such as correct bonding lengths, stable atomic environments, etc.

## Methods

### Conditional Sampling

The sampling process of diffusion models can be steered to yield outcomes with desired traits. Using classifier-free guidance techniques^26,27^ the model’s input ϵ_θ_(***x***_*t*_, *t*) → ϵ_θ_(***x***_*t*_, *t*, ***c***) is augmented by a vector-valued condition ***c*** = *c*(***x***_0_) that classifies certain features in the data ***x***_0_. During training, diffusion models learn to associate data ***x***_0_ with corresponding features *c*(***x***_0_), allowing trained models to generate samples ${\mathrm{x̂}}_{0}^{\left(T\right)}∼p\left(x|c^{\left(T\right)}\right)$ which exhibit desired target traits *c*(***x̂***_0_) ≈ ***c*** ^(T)^.

We keep the conditioning as simple as possible to retain the expressiveness of the diffusion model. To favor the sampling of compact atomic clusters, we define a radius *r*_cond_ around the origin in Euclidean space. For all cluster evolutions presented in this work, *r*_cond_ is chosen as the maximum span of the initial structure. Configurations in which all atoms lie within this sphere are marked as preferable, and the algorithm biases subsequent sampling toward similar structures. The preference is encoded through conditioning vectors assigned to all samples of the current population ***G***_τ_, which are then stored in the data buffer. After training, the population of the next generation can be sampled by the newly trained diffusion model $G_{\tau }$ , conditional to the target traits ***c*** ^(T)^.

To promote exploration, counteract possible mode collapse, and increase the likelihood of identifying a diverse set of high-fitness solutions even at high values for β, we employed novelty-conditional sampling^40^ during all evolutions. Novelty was quantified using a nonparametric *k*-nearest-neighbor entropy estimator,^73^ allowing the sampling process to favor structurally diverse configurations. The *k*-nearest neighbors are computed using the Euclidean (*L*_2_) metric, and novelty is defined as the logarithm of the mean distance after excluding the *k*-nearest neighbors, which allows repeated sampling of local regions before novelty penalization.

### Gradient Guidance

Conditioning the sampling process made it possible to produce relevant Lennard-Jones cluster configurations, even in the low-data regime of the initial generations. This can be attributed to the underlying potential,^47^ which allows particles that are positioned far apart from each other to interact and reorganize into physically reasonable structures. For the gold clusters, we instead use the medium MACE-OMAT-0 foundation model,^74^ which employs a finite cutoff radius in the message-passing procedure. As a result, atomic interactions vanish beyond a certain distance, and the subsequent relaxations often lead to fragmented aggregates rather than a single compact cluster.

To avoid this behavior, we propose gradient guidance. The approach introduces an auxiliary potential *U*(***x***) that biases the reverse diffusion process toward physically meaningful regions of configuration space. Rather than modifying the noisy latent variable ***x***_*t*_, the guidance is applied to the model’s denoised prediction, ***x̂***_0_(***x***_*t*_). At each step, this estimate of the final configuration is corrected by a gradient with respect to the auxiliary potential,

$${\mathrm{x̂}}_{0}^{\prime }\leftarrow {\mathrm{x̂}}_{0}-\eta \nabla_{{\mathrm{x̂}}_{0}}U\left({\mathrm{x̂}}_{0}\right)$$

where η is a scaling factor. Geometric and sterical constraints are defined in the physical coordinate space; applying them to ***x***_*t*_ would be ineffective, as the significant noise levels at large *t* obscure the geometric violations. By correcting ***x̂***_0_, we provide the model with a clean gradient signal. This allows us to actively guide the denoising trajectory, effectively steering the generative process toward the valid manifold. A visualization of this procedure is given in Figure 2.

**Figure 2:**
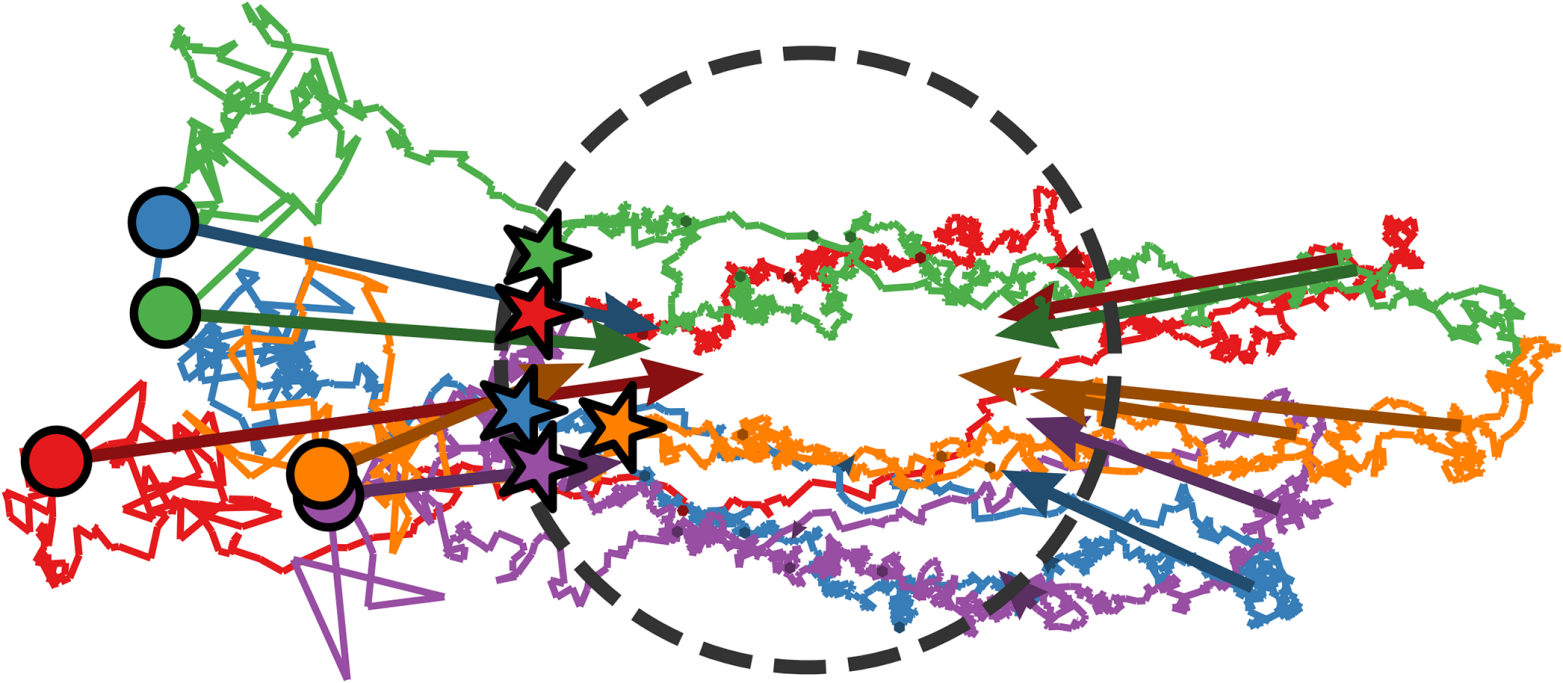
Visualization of geometric gradient guidance acting on diffusion trajectories. The plot shows the spatial denoising trajectory of five atoms initially sampled from a Gaussian distribution. Each path evolves from pure noise at *t* = *T* (solid circles) to an estimated clean configuration at *t* = 0 (star symbols). The model is untrained, thus the trajectories reflect only the influence of the gradient guidance term. At each step, the clean prediction ***x̂***_0_ is corrected by a gradient step with respect to the potential *U*(***x***), steering the atoms into the valid region. The dashed line marks the target radial boundary, while arrows (shown at two representative steps) indicate the applied guidance forces −∇*U* = −∇(*U*_overlap_ + *U*_geom_).

The auxiliary potential *U*(***x***) consists of two contributions. The first term, *U*_overlap_, enforces short-range repulsion by penalizing interatomic distances below a threshold *d*_min_,

$$U_{\mathrm{overlap}}\left(x\right)=\underset{i<j}{\sum }{[\max(0,d_{\min}-d_{ij})]}^{2}$$

The second term, *U*_geom_, constrains the global shape of the generated structures and prevents fragmentation. We employ a radial potential that penalizes atoms lying outside a spherical region of space defined by a radius *R*_out_,

$$U_{\mathrm{geom}}\left(x\right)=\underset{k}{\sum }{[\max(0,∥x_{k}∥-R_{\mathrm{out}})]}^{p}$$

where *p* is a user-defined power factor controlling the steepness of the penalty. Similarly, an ellipsoidal variant allows the generation of anisotropic structures by normalizing the atomic coordinates by the ellipsoid radii ***r*** = (*r*_*x*_, *r*_*y*_, *r*_*z*_) before computing the norm.

Gradient guidance alone acts only at inference and can be inefficient if the base model frequently predicts physically invalid structures. Therefore, we augment the modified loss *L*_evo_(θ) in eq 3 with the total potential *U*(***x̂***_0_) evaluated on the model’s clean data prediction,

$$L_{\mathrm{reg}}\left(\theta \right)=L_{\mathrm{evo}}\left(\theta \right)+\lambda_{\mathrm{reg}}E_{x_{0},t,\epsilon }U\left({\mathrm{x̂}}_{0}\left(x_{t}\right)\right)$$

where λ_reg_ controls the regularization strength. Penalizing violations of steric or geometric constraints at the level of ***x***_0_ embeds these constraints directly into the learned score function, effectively teaching the model to generate compact and nonoverlapping configurations.

The influence of *U*_overlap_ is visible in Figure 2, where the orange and purple atoms are initially sampled closer than *d*_min_. As a result, the guidance vectors diverge instead of pointing orthogonally toward the target boundary. With this improvement, virtually all sampled configurations are complete clusters during the whole evolution. The only samples consisting of smaller clusters are artifacts of the foundation models, which can rarely assign very low energy values to unphysical structures during the relaxation process. These are penalized by the algorithm and are not chosen for training.

### Relaxation and Evaluation

The relaxations were performed by the FIRE optimizer^75^ implemented in the Atomic Simulation Environment (ASE).^76^ The maximum forces after relaxation were set to *f*_max_ = 0.001 eV/Å. In the case of the Lennard-Jones clusters, we used the well-known LJ potential^47^ for the evaluation of the energies of the sampled configurations, an analytical two-body potential that separates the interaction between atoms into a repulsive and an attractive part. For the neutral gold clusters, we use the medium MACE-OMAT-0 model (in the following abbreviated as OMAT),^74^ a machine-learning foundation model^77^ based on the MACE^78^ architecture. Rarely, OMAT assigns very low energies to unphysical samples during the relaxation procedure, disturbing the ordering of the fitness. To prevent this behavior, fitness values of configurations with energies lower than a defined threshold are multiplied by a penalty factor.

### Training

We used a feed-forward multilayer perceptron implemented in PyTorch^79^ as the neural network for the diffusion model. It contained 96 hidden units in 8 hidden layers each and used the rectified linear unit (ReLU) as the activation function. The input of the network is the raw Cartesian coordinates of the configurations. The number of noising and denoising steps was set to 5000 with a linear alpha schedule. The number of training steps for the diffusion model at the end of each generation was chosen as 1000. The training was optimized by ADAM^80^ with a learning rate of 10^–2^ and a weight decay of 10^–5^. The buffer size of the successively increasing data set was set to 1000. Configurations from the buffer were selected for training via roulette wheel selection with a crossover ratio of 0.125 and a mutation rate of 0.05.^40^

### System-Specific Parameters

The roulette wheel selection mechanism weights the fitness of the samples according to eq 4. The parameter β was set to 20.0 for the Lennard-Jones clusters to emphasize the selection of configurations with high fitness. For the gold clusters, we compared three values, namely 20.0 as reference, 2.0 for more evenly distributed selection, and 5.64, which corresponds to a temperature of approximately 2000 K. All LJ cluster evolutions were executed in reduced units by setting *ε*_LJ_ = σ_LJ_ = 1 and started with randomized clusters as founders. The population size was set to λ = 64 for all LJ cluster evolutions and λ = 256 for all gold cluster evolutions. The initial step sizes for CMA-ES and the adaptive diffusion strategy were chosen as σ^init^ = 0.3 and σ^init^ = 1.5, respectively. During the diffusion evolutions, all generated individuals were relaxed until all forces were lower than *f*_max_ = 0.001 eV/Å. For better comparison, the CMA-ES populations were relaxed in the same fashion one time after the last generation.

### Dimensionality Reduction

This section describes the workflow of the visualization of the visited areas in configuration space in Figure 4 and Figure S1. First, the positions of the atoms in the sampled configurations were encoded into spherical Bessel descriptors as implemented in NeuralIL^81^ to yield representations of the atomic environments that are translational and rotational invariant. The parameter *n*_max_ was set to 5 (which controls the number of basis functions), and the cutoff radius to *r*_cut_ = 4.0 Å. The resulting local descriptors (in our case vectors with 21 entries each for every atom) were summed up over all atoms of a given configuration to obtain the global descriptors. These descriptors were then reduced to two dimensions with the help of the uniform manifold approximation and projection (UMAP) algorithm.^82^ The number of neighbors was set to 100 and the minimum distance between the data points to 1.0. The distances were computed with the correlation metric.

### Computational Cost

The number of energy force evaluations can be estimated by *n*_eval_ = *n̅*_steps_ · λ · *n*_gen_ where *n̅*_steps_ is the average number of steps used for the structure relaxations, λ is the population size, and *n*_gen_ is the number of generations of the evolution. For all calculations, we chose a tight maximum force of *f*_max_ = 0.001 eV/Å as the termination criterion for the relaxation cycle. While the exact number of relaxation steps depends on the sampled structures, most relaxations required 100 – 500 iterations, and we take *n̅*_steps_ = 300 evaluations as a representative value.

For the LJ_31_ cluster (λ = 64), the global minimum was found after *n*_gen_ = 141 generations, corresponding to approximately *n*_eval_ ≈ 2 · 10^6^ evaluations in total. For Au_31_ we increased the population size to λ = 256. The lowest energy cluster was found after *n*_gen_ = 14 generations, corresponding to approximately *n*_eval_ ≈ 1 · 10^6^ force evaluations in total. The tight force cutoff does make the relaxation a computational bottleneck of the algorithm. More moderate convergence criteria, such as *f*_max_ = 0.01 eV/Å, often yield comparable results while substantially reducing the computational cost.

## Results

### Lennard–Jones Clusters

We use Lennard-Jones clusters ${\mathrm{LJ}}_{n_{\mathrm{atom}}}$ containing between *n*_atom_ = 5 and *n*_atom_ = 40 atoms as a benchmark system to visualize the differences between the CMA-ES and the adaptive diffusion strategy. The results can be seen in Figure 3. They underline the strength of the adaptive diffusion for higher-dimensional optimization problems. In agreement with earlier results for a similar algorithm,^14^ CMA-ES was able to find the global minima for clusters only up to around *n*_atom_ = 17, depending on the initial random seed. The diffusion discovered the global minima for all investigated clusters with an energy difference Δ*E*_LJ_ lower than 10^–6^ eV. While all CMA-ES evolutions were executed for 1000 generations, the adaptive diffusion strategy was able to converge to the corresponding global minima using a smaller number of generations. Global minima for clusters smaller than *n*_atom_ = 25 were always found in less than 10 generations, and larger clusters, in all cases except LJ_34_ and LJ_38_ in less than 150 generations. The point we want to emphasize is the ability of the diffusion to find all global minima with the same random seed in a single evolution per cluster. This means that the adaptive diffusion strategy does not rely on large numbers of evolutions with different random seeds to yield useful results.

**Figure 3:**
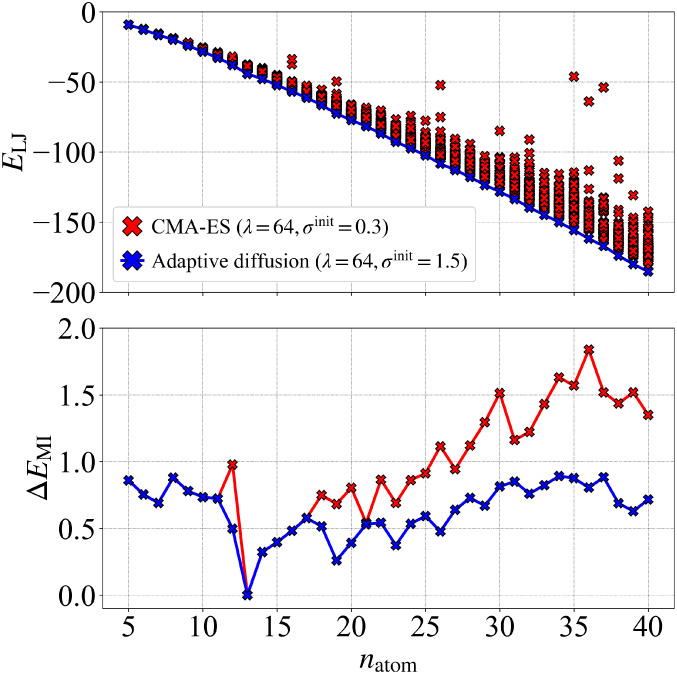
Comparison between CMA-ES and adaptive diffusion for Lennard–Jones clusters. **(Top)** The lowest energies *E*_LJ_ from the last generation for Lennard–Jones clusters containing *n*_atom_ ∈ {5, ..., 40} atoms. For every cluster, one adaptive diffusion evolution and 50 CMA-ES evolutions were executed to yield a distribution of final energies. **(Bottom)** The normalized energy differences $\Delta E_{\mathrm{MI}}=\left|E_{\mathrm{LJ}}-E_{\mathrm{MI}}\right|/\sqrt{n_{\mathrm{atom}}}$ between the LJ evolution energies and *E*_MI_, a function fitted to the energies of the first four complete Mackay icosahedra with *n*_atom_ ∈ {13, 55, 147, 309}, which are particularly stable.^83^

In addition to it becoming increasingly challenging to locate the global minimum as cluster sizes grow, larger LJ clusters also exhibit a vast number of competing local minima corresponding to structurally distinct configurations with nearly degenerate energies. While smaller clusters such as LJ_13_ often feature connectivity graphs with one dominant global minimum structure (in this case, a complete Mackay icosahedron), larger clusters tend to form diverse structures.^83^ For such systems, systematically mapping out the ensemble of low-lying minima can be as important as identifying the global minimum itself.

Within the adaptive diffusion strategy, the parameter β in the fitness mapping, eq 4, controls the selection pressure and, therefore, the balance between exploration and exploitation of competing basins. In contrast, CMA-ES lacks an intrinsic mechanism to promote and maintain diversity across basins and typically requires repeated restarts in the hope of discovering alternative solutions. We illustrate these differences in terms of two well-known LJ cluster sizes, LJ_31_ and LJ_38_, which represent two distinct and challenging cases for any optimization algorithm.^47^

Great attention has also been given to the LJ_38_ cluster because it exhibits a characteristic double-funnel energy landscape with a large energy barrier.^84,85^ The global minimum corresponds to a face-centered-cubic truncated octahedron^86,87^ and the second-lowest energy minimum is an incomplete Mackay icosahedron.^88^ As a result, LJ_38_ represents a small area of stability, see Figure 3, and features three distinct low-energy clusters originating from two competing funnels.^85^

Figure 4a shows a two-dimensional UMAP embedding of the global descriptors (one 21-dimensional vector per structure) obtained during the evolution of the LJ_38_ cluster. The descriptors corresponding to the three lowest-energy minima identified by the adaptive diffusion strategy are highlighted by white markers and form distinct islands away from the main descriptor distributions. These isolated regions represent characteristic geometric features of stable configurations. The absence of CMA-ES descriptors in these regions highlights its limited exploration capabilities for high-dimensional, multimodal optimization problems.

**Figure 4:**
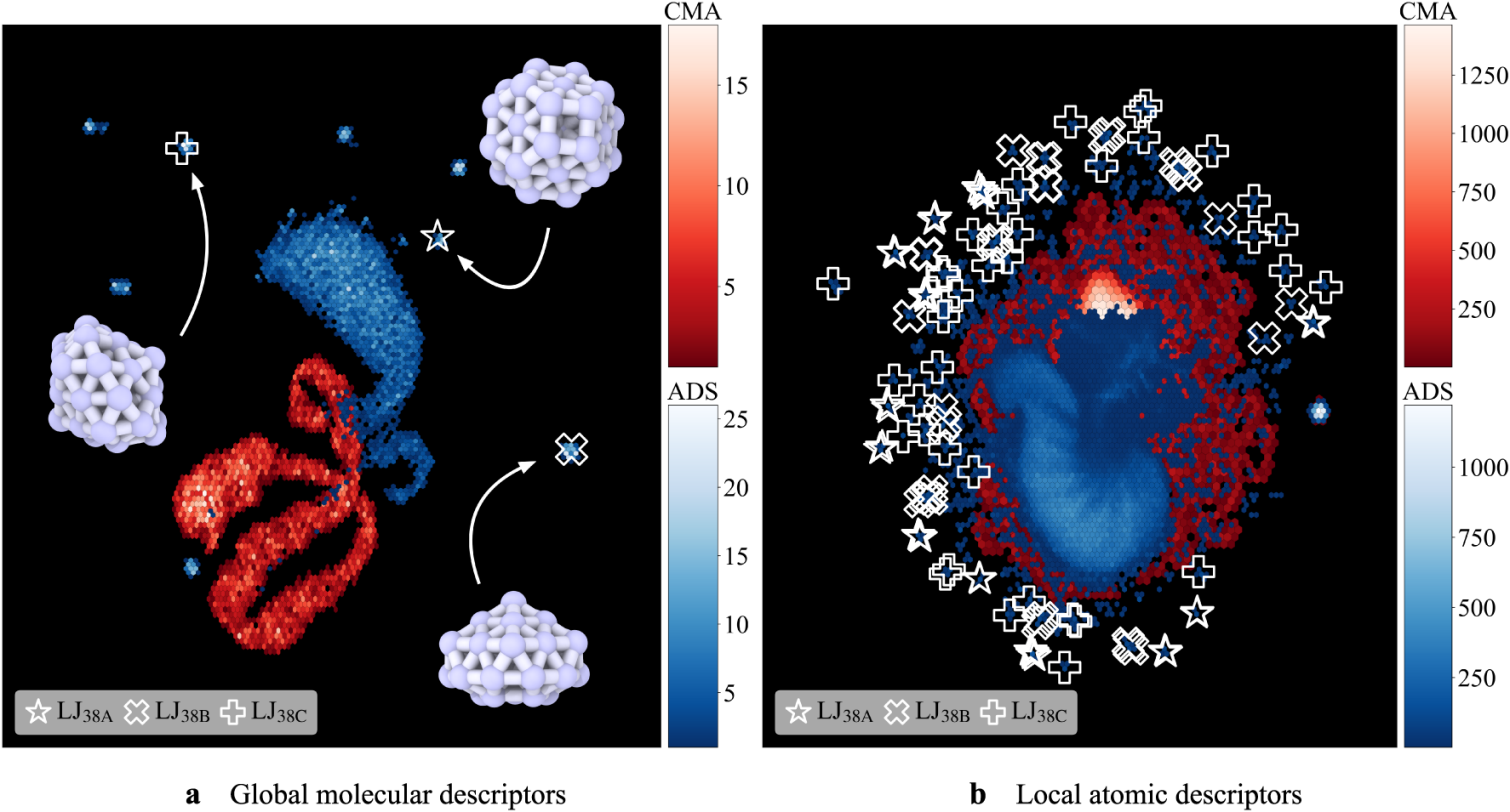
UMAP of LJ_38_ cluster with global and local descriptors. Both figures show the UMAP of the CMA-ES (red) and adaptive diffusion (blue) evolutions with a randomized LJ_38_ cluster as the founder structure. The population sizes were chosen as λ = 64 and the initial step sizes as σ^init^ = 0.3 and σ^init^ = 1.5, respectively. Both evolutions ran for 1000 generations, from which 100 generations (every 10th generation) were chosen for the UMAP visualization. The left panel **a** shows the UMAP of the global descriptors (one for every structure), while the right panel **b** shows the local descriptors (one for every atom). Highlighted with white markers are the three minima LJ_38A_, LJ_38B_ and LJ_38C_ with the lowest known energies stated in Doye et al.,^85^ for every minimum, either one global descriptor in panel **a** or 38 local descriptors in panel **b**.

Figure 4b shows the local descriptors (one 21-dimensional vector per atom) for the same evolutions. As for the global descriptors, the local descriptors (38 for every structure) of the three lowest minima are indicated by white markers. These results support the insights from Figure 4a, namely that the local descriptors are located on islands outside the main descriptor distributions, again not accessible to CMA-ES.

The LJ_31_ cluster belongs to a group of clusters with relatively low stability, see Figure 3. Rather than being dominated by one or two deep funnels, the landscape is characterized by a large number of competing minima. This topology can be rationalized by viewing the system as 18 atoms distributed around a complete LJ_13_ Mackay icosahedron.^89^ While the 13-atom core represents a strongly stabilizing motif, the many possible ways of decorating this core with the remaining atoms give rise to a multitude of shallow subfunnels and local minima. UMAP visualizations for the LJ_31_ cluster can be found in Figure S1 in the Supporting Information.

To understand how the adaptive diffusion strategy explores this complex landscape, we analyze the connectivity of the sampled structures, defined as the number of atoms that are bonded into a single cluster. The resulting connectivity histograms for LJ_31_ are shown in Figure 5. In the first generation (left panel), the sampled configurations are widely dispersed across configuration space and include fragmented structures composed of multiple small clusters. The largest cluster observed at this stage contains 30 atoms, with one atom remaining detached.

**Figure 5:**
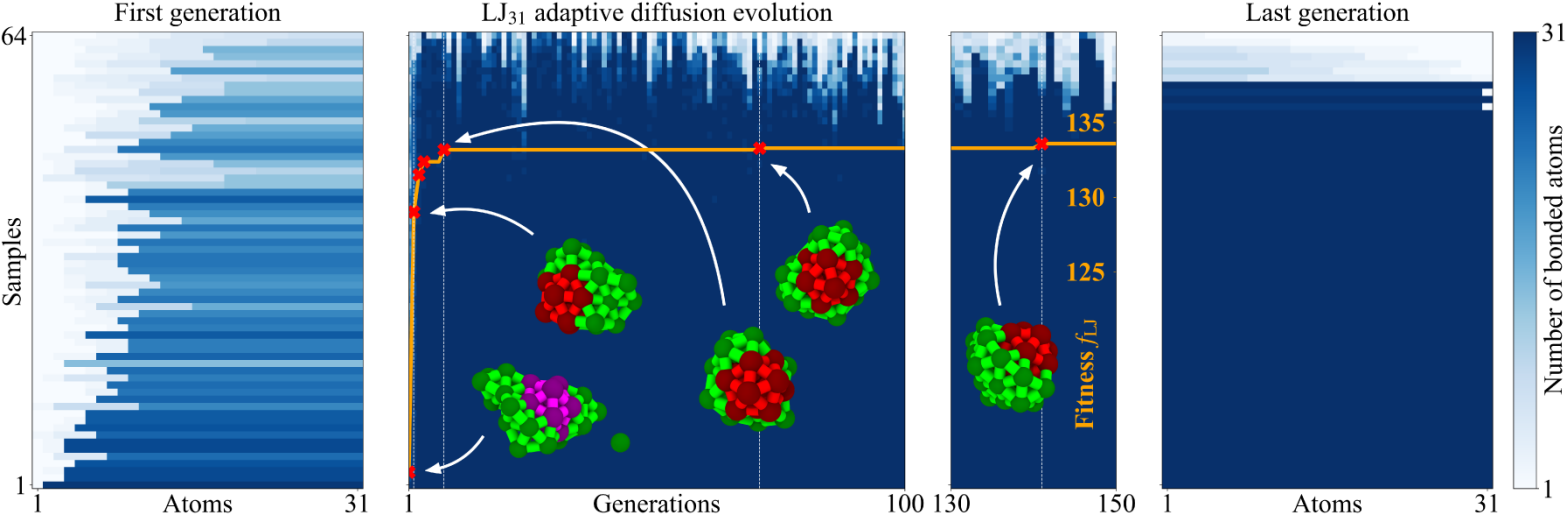
Histogram of LJ_31_ cluster connectivity. **(Left)** The figure depicts the first generation of the LJ_31_ adaptive diffusion evolution, consisting of 64 samples with 31 atoms each. Every row represents one sample, where the colors encode the connectivity of the atoms. Atoms that are bonded together are illustrated by the same color, the edge cases being white for single atoms and blue for complete LJ_31_ clusters (see the color bar on the right). **(Right)** The figure depicts the same information but for the last generation of the evolution. **(Center)** The figure plots the largest clusters from all 64 samples in one column for every generation. Additionally, the highest fitness *f*_LJ_ for every generation is plotted as an orange line, where generations with increasing fitness are indicated by red markers. Selected structures at interesting points in the evolution are displayed as well. The pink- and red-colored close-packed cores at the center of the structures consist of 12 and 13 atoms, respectively. The red-colored core represents a complete Mackay icosahedron and is equivalent to the global minimum of the LJ_13_ cluster.^89^

As the evolution proceeds, the diffusion model increasingly concentrates probability mass in regions corresponding to lower energies, that is, deeper parts of the landscape. By the final generation (right panel), most samples correspond to compact clusters in which nearly all atoms are bonded together, indicating that the model has learned to preferentially sample configurations where the atoms are close to each other, reflecting that such configurations yield the highest fitness values, *f*_LJ_ = −*E*_LJ_.

Importantly, the sampling does not collapse onto a single funnel. Even in the later generations (center panel of Figure 5), the diffusion model continues to generate configurations with smaller clusters and even isolated atoms. Although these structures are associated with higher energies, their persistent presence indicates that the algorithm maintains access to higher-lying regions of the landscape. This behavior is essential for avoiding premature trapping in suboptimal funnels and enables transitions between competing low-energy basins. These results further support the claim of high diversity in the sampling process and the exploratory capability of the algorithm.

The evolution of the best fitness values, *f*_LJ_, as shown in Figure 5, and the corresponding structures for specific generations where the maximum *f*_LJ_ increases, are consistent with this interpretation. During the first 10 generations, rapid improvements indicate a descent from high-energy regions into several low-energy funnels. This initial phase is followed by long intervals with no improvement in fitness. It should be emphasized, however, that during these intervals, the diffusion model continues to learn by sampling additional structures, eventually enabling the sampling of better configurations until the global minimum is found.

The visualizations of the selected structures in Figure 5 provide insights into the inner workings of the algorithm. Focusing on the close-packed atom cores highlighted in pink and red, it can be seen that a complete 13-atom Mackay icosahedron already emerges in the second generation and is maintained throughout the evolution. Following the emergence of this stable core, optimization primarily involves the arrangement of the remaining 18 atoms decorating the close-packed core.

This behavior indicates that the diffusion-based framework rapidly learns fundamental low-level structural features of the PES, such as the tightly packed centerpiece. More complex and detailed high-level features, including the optimal surface structure and competing decorations of the given cluster, are then resolved over extended evolutionary time scales.

By conceptually associating adaptive diffusion generations with the denoising steps of traditional diffusion models, this separation of time scales can be related to prior work on feature hierarchies during denoising. Studies conducted on image diffusion models have shown that low-level features persist much longer than high-level features.^90,91^ Another perspective is provided by interpreting the mechanisms that lead to these phase transitions as part of a spontaneous symmetry-breaking framework, effectively dividing the generative dynamics into distinct phases.^92^ The present results suggest that an analogous hierarchy governs diffusion-driven PES optimization.

A similar visualization of the connectivity for the LJ_38_ cluster can be found in Figure S2 in the Supporting Information.

### Gold Clusters

To assess the adaptive diffusion strategy under more realistic and chemically complex conditions, we extend our analysis to the neutral gold clusters. In contrast to LJ clusters, gold clusters exhibit pronounced directional bonding and a PES with qualitatively different low-energy motifs. Rather than forming the same stable core behavior as, e.g., the LJ_31_ clusters, the larger gold clusters display a rich variety of low-energy motifs, including fcc-like pyramidal, shell-like flat-cage, spherical-like hollow-cage, tubular cigar-shaped, and stuffed amorphous structures.^51,52,58–65^ This structural diversity makes gold clusters a challenging test case for any global optimization algorithm.

For reference, we use the gold cluster configurations found in the Quantum Cluster Database (QCD).^93^ QCD contains a large number of clusters taken from the literature or found via genetic-algorithm searches. As examples, we choose the Au_13_, Au_31_ and Au_38_ cluster sizes. For each size, we extract the lowest-energy configurations, resulting in 30 structures for Au_13_ and Au_31_, and 28 for Au_38_. The relative stability of gold cluster isomers is known to depend sensitively on the choice of density functional.^94^ The goal of this study is not to establish a definitive energetic ordering but rather to evaluate the ability of the adaptive diffusion strategy to explore and navigate a complex PES. We therefore reoptimize the QCD gold clusters using the same OMAT foundation model that is employed during diffusion-based sampling. This ensures a consistent reference set of structures for evaluating the diffusion model. Visualizations of the 15 most stable OMAT_*m*_ structures for Au_13_, Au_31_ and Au_38_ can be found in Figure S3, Figure S4 and Figure S5 in the Supporting Information.

The energies of these relaxed structures were then compared to configurations obtained by diffusion-driven optimizations executed for 125 generations with a population size of λ = 256 and varying evolutionary pressure β and gradient-based guidance. The number of generations necessary to find such structures for the first time was recorded. Plots for all evolutions can be found in Figure S6, Figure S7 and Figure S8 in the Supporting Information, while a cumulative overview is given in Figure 6. In all three cases, the 15 lowest energy structures were found. For the small Au_13_ cluster, only a few generations were needed, whereas for the larger Au_31_ and Au_38_ clusters, the diffusion is still identifying new structures after 100 generations.

**Figure 6:**
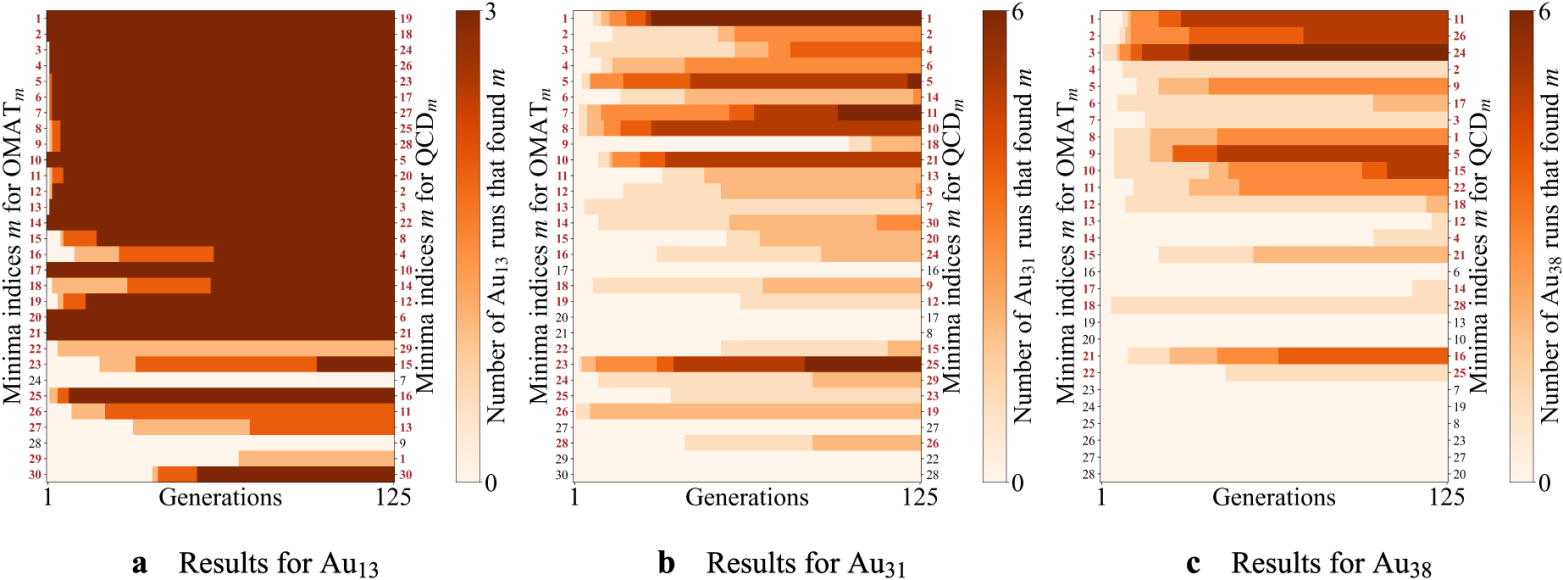
Comparison between OMAT-relaxed QCD minima and results of an adaptive diffusion evolution. Shown are the indices *m* of Au_13_, Au_31_ and Au_38_ QCD cluster minima, which have energies equivalent to structures found by the adaptive diffusion strategy (up to a difference of Δ*E*_OMAT_ = 10^–5^ eV) using the OMAT foundation model. The plots show in which generation these structures were found first. Every plot combines the results of 3 (for Au_13_ in the left panel **a**) and 6 (for Au_31_ in the center panel **b** and Au_38_ in the right panel **c**) evolutions with different input parameters. The colors indicate the number of different evolutions that found the same minimum *m* (see color bars on the right). Structures that were not found are kept white during the whole evolution. The left axes depict the minima indices *m* as ordered by the OMAT foundation model, and the right axes as ordered by the QCD database, in which colored indices identify structures that were found at least once.

While OMAT leads to a reordering of isomer energies relative to the QCD database, no qualitatively new structural motifs were generated. This indicates that although the foundation model changes the relative energy ordering, the underlying topology of the PES, specifically the set of relevant low-energy basins, remains essentially unchanged. In the following, minima are labeled as QCD_*m*_ or OMAT_*m*_ depending on whether they are ordered by QCD or OMAT energies, where *m* denotes the index of the minimum. As illustrated by all three panels of Figure 6, the two orderings differ quite substantially. For the Au_13_ clusters, for example, QCD_1_ corresponds to OMAT_29_, while OMAT_1_ corresponds to QCD_19_.

Despite the reordering, the adaptive diffusion strategy consistently recovers the five most stable QCD structures for all three cluster sizes. This highlights a key advantage over global optimization approaches that focus narrowly on locating the global minimum. When the energy model changes, such methods typically require a complete restart. In contrast, the diffusion-based framework naturally samples a structurally diverse ensemble of candidates that can be reevaluated under different energy functionals without repeating the search process.

An adaptive diffusion evolution for the Au_31_ cluster is shown in Figure 7. The two lowest-energy hollow structures are identified within fewer than 60 generations. Comparison with the QCD database further shows that the diffusion reliably recovers not only the lowest-energy structures but also a broad set of higher-lying local minima. In the run illustrated in Figure 7, 18 of the 30 QCD Au_31_ reference structures are identified. The eight most stable structures among these, each corresponding to a cage-like structure, are indicated by yellow markers at the generation where they first appear. Across the six independent runs performed for Au_31_, the diffusion recovers nine out of the ten original lowest-energy Au_31_ QCD structures, despite working on a different PES (see Figure 6b). This robustness highlights the intrinsic structural diversity maintained by the diffusion-based optimization.

**Figure 7:**
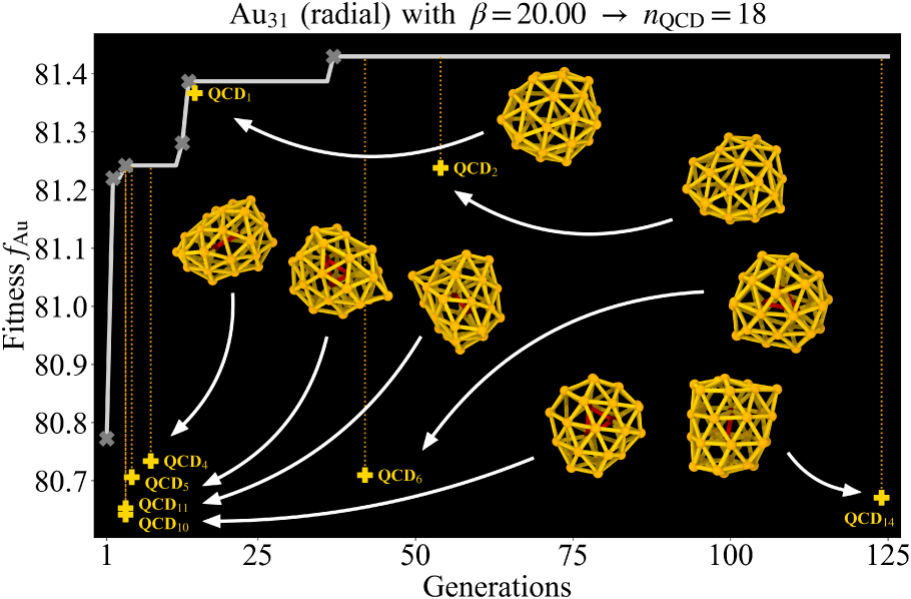
OMAT-relaxed QCD minima found during an adaptive diffusion evolution. Plotted are the maximum fitness values *f*_Au_ on a gray line for every generation of the adaptive diffusion evolution for the Au_31_ gold cluster. The eight most stable structures (as ordered by OMAT) out of all 18 found configurations that exhibit energies equivalent to the OMAT-relaxed QCD*_m_* minima with *m* ∈ {1, 2, 4, 6, 5, 14, 11, 10} are indicated by yellow markers at the generation where they were found first (see Figure S7d for comparison). The height of the markers denotes the fitness values *f*_Au_ of the clusters calculated by OMAT. The configurations are also visualized, where gold atoms placed inside the hollow or stuffed cage structures (none for QCD_1,2_, two for QCD_4,14,11_ and three for QCD_6,5,10_) are colored red.

QCD_11_, QCD_10_, QCD_5_ and QCD_4_ were already found in the first 10 generations. While the maximum fitness *f*_Au_ is increasing during the evolution, minima such as QCD_1_, QCD_6_ and QCD_2_ were found at generations 14, 41, and 53, respectively. Finally, QCD_14_ was found much later at generation 123. Especially QCD_14_, but also the other minima found in the evolution, including the 10 QCD_*m*_ configurations not shown in Figure 7 (see Figure S7d for comparison), demonstrate the ability of the adaptive diffusion strategy to continuously generate diverse configurations throughout the entire evolution, even up to the last generation. This observation holds true even though the QCD_*m*_ minima exhibit lower fitness values than the maximum fitness samples found up until this point (depicted as the vertical difference between the gray *f*_Au_ line plot and the yellow QCD_*m*_ markers in Figure 7), emphasizing the effect of the novelty conditioning technique.^40^

The found configurations confirm a different stacking mechanism for gold clusters than for the Lennard-Jones clusters. Stable structures containing approximately 30 gold atoms exhibit hollow or stuffed cage configurations, where almost all atoms are part of the outer surface. Even though there would be room inside the cages for a small number of atoms, this space is not always filled. Most of the time, either two or three gold atoms are positioned inside, while still displaying small pockets of empty space, where the distances between the center atoms and the outer shell are larger than the typical bond length. Sometimes, there are no gold atoms positioned inside the empty shell, as it is the case for QCD_1_ and QCD_2_. This leads to the conclusion that gold clusters of this size do not form close-packed cores such as LJ_31_ (see Figure 5 for comparison), but instead tend to shape into amorphous structures with complex surface geometries.

## Conclusion

We adapted an adaptive diffusion strategy for structural exploration and extended it by two key mechanisms: physics-informed guidance and local relaxation to ensure effective convergence.

Instead of using diffusion as a data-generation tool inside a one-time training procedure, the algorithm utilizes a series of progressively refined diffusion models as part of an evolutionary search strategy. In contrast to heuristic evolution strategies such as CMA-ES, the strategy is well suited for high-dimensional problems and is able to sample large amounts of high-quality configurations in parallel. The application to Lennard-Jones clusters showed that a large number of local and global minima for LJ_31_ and LJ_38_ clusters were found in a single evolution per system. An extended analysis of the sampled Au_13_, Au_31_ and Au_38_ neutral gold clusters further emphasized the ability of the algorithm to explore metastable configurations alongside the global minimum.

The adaptive diffusion strategy demonstrates how the representational power of diffusion models can be embedded within an evolutionary search framework. The effectiveness of the present lightweight implementation on high-dimensional structure optimization problems highlights the potential of the approach. At the same time, casting the search process as a learning problem opens the door for systematically incorporating advances from the machine-learning literature, providing a clear path toward further improvements.

One direction is the refinement of the conditioning mechanisms. More involved criteria could be incorporated to steer the search toward structures with desired properties. For instance, features derived from experimental data could be used to bias the search toward structures exhibiting system-specific target features. The novelty-conditioning module could likewise be improved by replacing the current distance metric with one based on atomic descriptors, which provide a more meaningful notion of structural diversity. More sophisticated neural network architectures, either augmented with attention mechanisms^95–97^ or designed as equivariant graph neural networks,^98–101^ could enhance the quality of the generated structures. Refined denoising mechanisms, such as flow matching,^102–104^ may provide smoother and more stable generation trajectories, potentially leading to higher-quality configurations. Finally, the current functional form of the mapping *h*[*f*(***x***)] allocates large weights to higher fitness values. While advantageous for finding the most stable structures, it can hinder exploration when the objective is to characterize multiple local minima. A possible alternative could be to use stepwise functions, which would push the selection mechanism toward these configurations.

## Supplementary Material



## Data Availability

Data and software availability: Selected evolution and structure files containing results presented in this paper are available on Zenodo at 10.5281/zenodo.19001618. Our code is based on the CondEvo package, which is available on GitHub at https://github.com/bhartl/CondEvo.

## Notes

Additional figures including UMAP, connectivity, structure and exploration plots. (PDF)
