# Supplementary material for: Guided Adaptive Diffusion: An Evolutionary Framework for Multimodal Atomistic Structure Prediction
Source: J Chem Inf Model. 2026 Jun 25;66(13):7414–25. doi: 10.1021/acs.jcim.6c00843 (PMC13370864; doi:10.1021/acs.jcim.6c00843)
Supplement: Supplementary file 1 [file ci6c00843_si_001.pdf]

Supplementary material for *Guided adaptive diffusion: An evolutionary framework for multi-modal atomistic structure prediction*

Alexander Adel[1], **Jakub Szmitek**[1], **Benedikt Hartl**[2,3],
Ralf Wanzenböck[1] and **Georg K. H. Madsen**[1]*

[1] Institute of Materials Chemistry, TU Wien, 1060 Vienna, Austria

[2] Allen Discovery Center at Tufts University, 02155 Medford, MA, USA

[3] Institute of Theoretical Physics, TU Wien, 1040 Vienna, Austria

*E-mail: georg.madsen@tuwien.ac.at

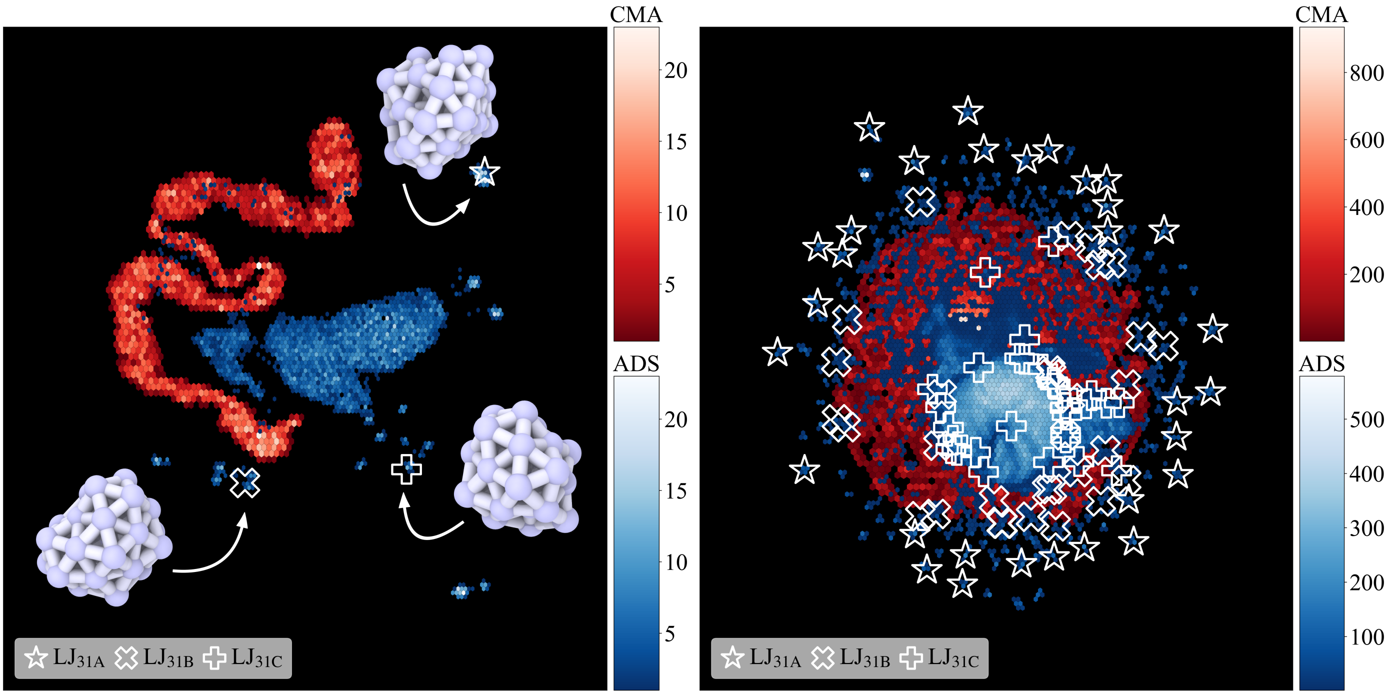

a Global molecular descriptors **b** Local atomic descriptors

Figure S1 – UMAP of LJ_{31} cluster with global and local descriptors. Both figures show the UMAP of the same CMA-ES (red) and adaptive diffusion (blue) evolutions with a randomized LJ_{31} cluster as founder structure. The population sizes were chosen as $\lambda = 64$ and the initial step sizes as $\sigma^{\text{init}} = 0.3$ and $\sigma^{\text{init}} = 1.5$, respectively. Both evolutions ran for 1000 generations, from which 100 generations (every 10th generation) were chosen for the UMAP visualization. The left figure **a** shows the UMAP of the global descriptors (one for every structure), while the right figure **b** shows the local descriptors (one for every atom). Highlighted with white markers are the three minima LJ_{31A}, LJ_{31B} and LJ_{31C} with the lowest known energies stated in Calvo et al.,[46] for every minimum either one global descriptor in **a** or 31 local descriptors in **b**.

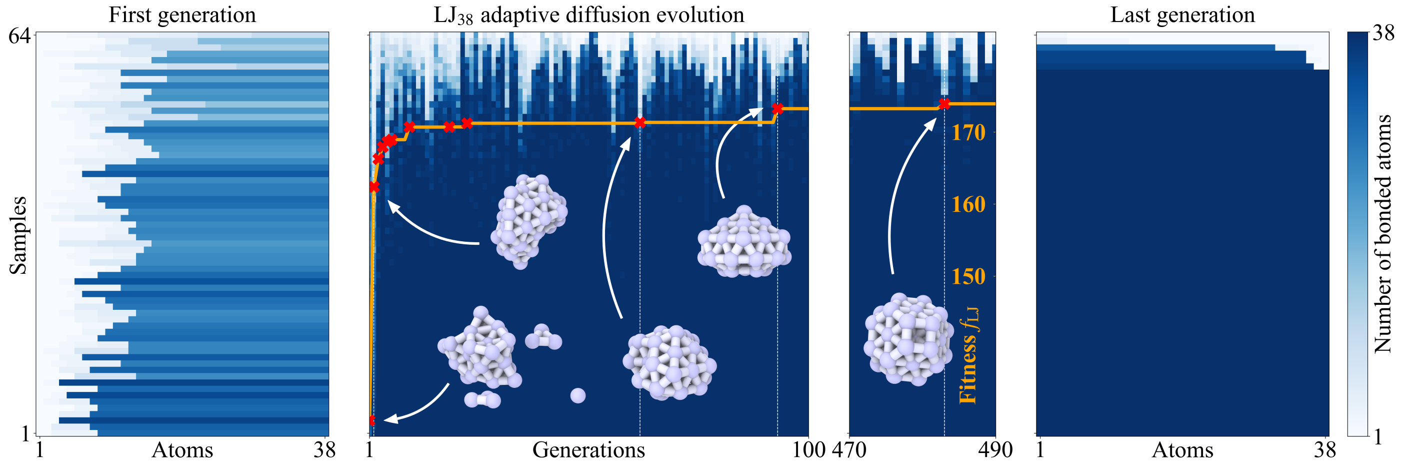

Figure S2 – Histogram of LJ_{38} cluster connectivity. **(Left)** The figure depicts the first generation of the LJ_{38} adaptive diffusion evolution, consisting of 64 samples with 38 atoms each. Every row represents one sample, where the colors encode the connectivity of the atoms. Atoms that are bonded together are illustrated by the same color, the edge cases being white for single atoms and blue for complete LJ_{38} clusters (see the color bar on the right). **(Right)** The figure depicts the same information, but for the last generation of the evolution. **(Center)** The figure plots the largest clusters from all 64 samples in one column for every generation. Additionally, the highest fitness f_{LJ} for every generation is plotted as an orange line, where generations with increasing fitness are indicated by red markers. Selected structures at interesting points in the evolution are displayed as well.

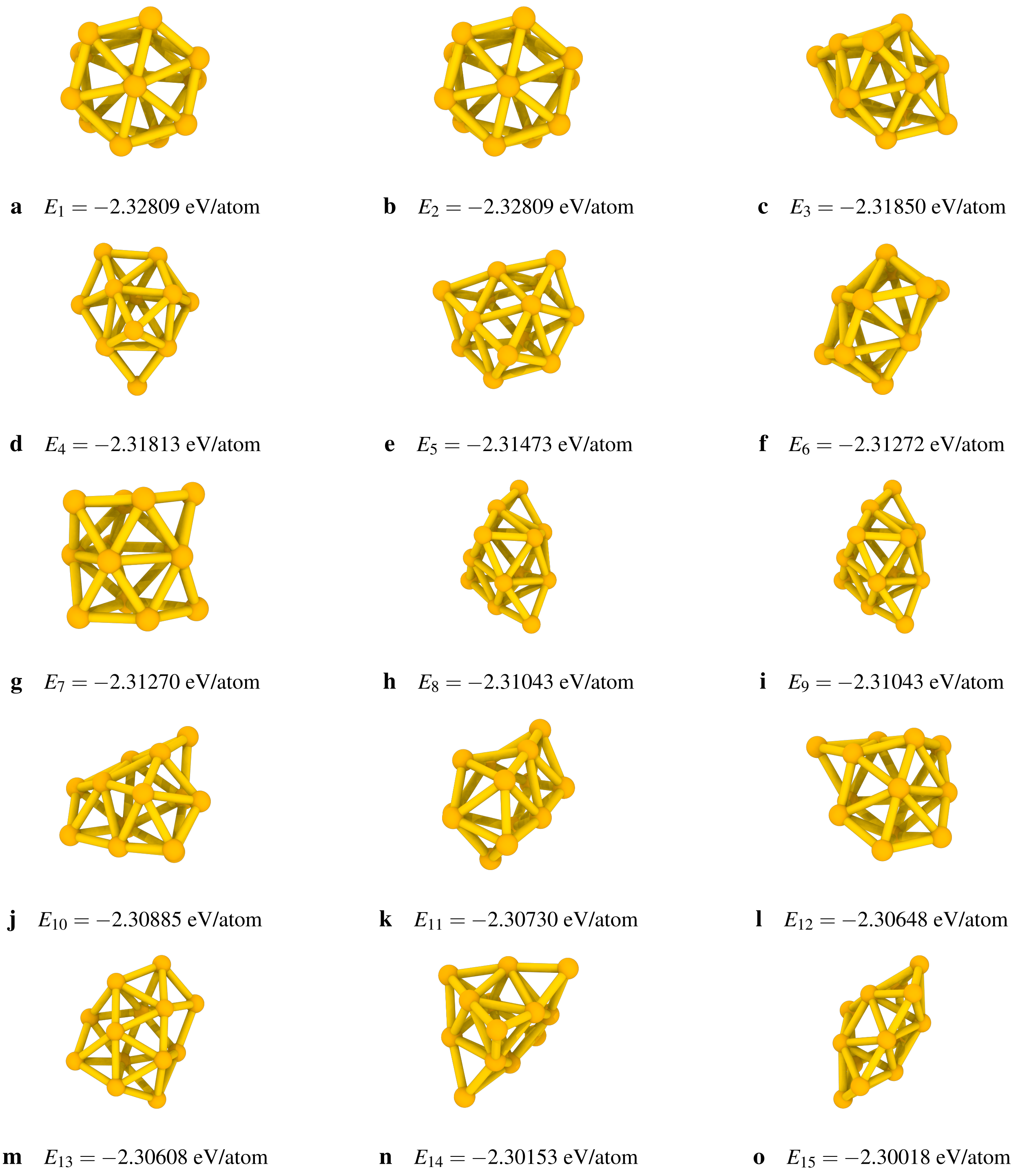

Figure S3 – Top 15 OMAT$_m$ Au$_{13}$ clusters. Shown are visualizations of OMAT$_m$ with $m \in \{1, \ldots, 15\}$. These are the 15 most stable Au$_{13}$ clusters found by the adaptive diffusion evolution. Also given are the energies per atom E_m for every OMAT$_m$ structure. Rarely, OMAT relaxes two energetically very similar QCD structures to the same configuration, one example being the pair OMAT$_1$ and OMAT$_2$ for Au$_{13}$, where the two corresponding original QCD structures exhibit a DFT energy difference of only 0.02 eV.[93]

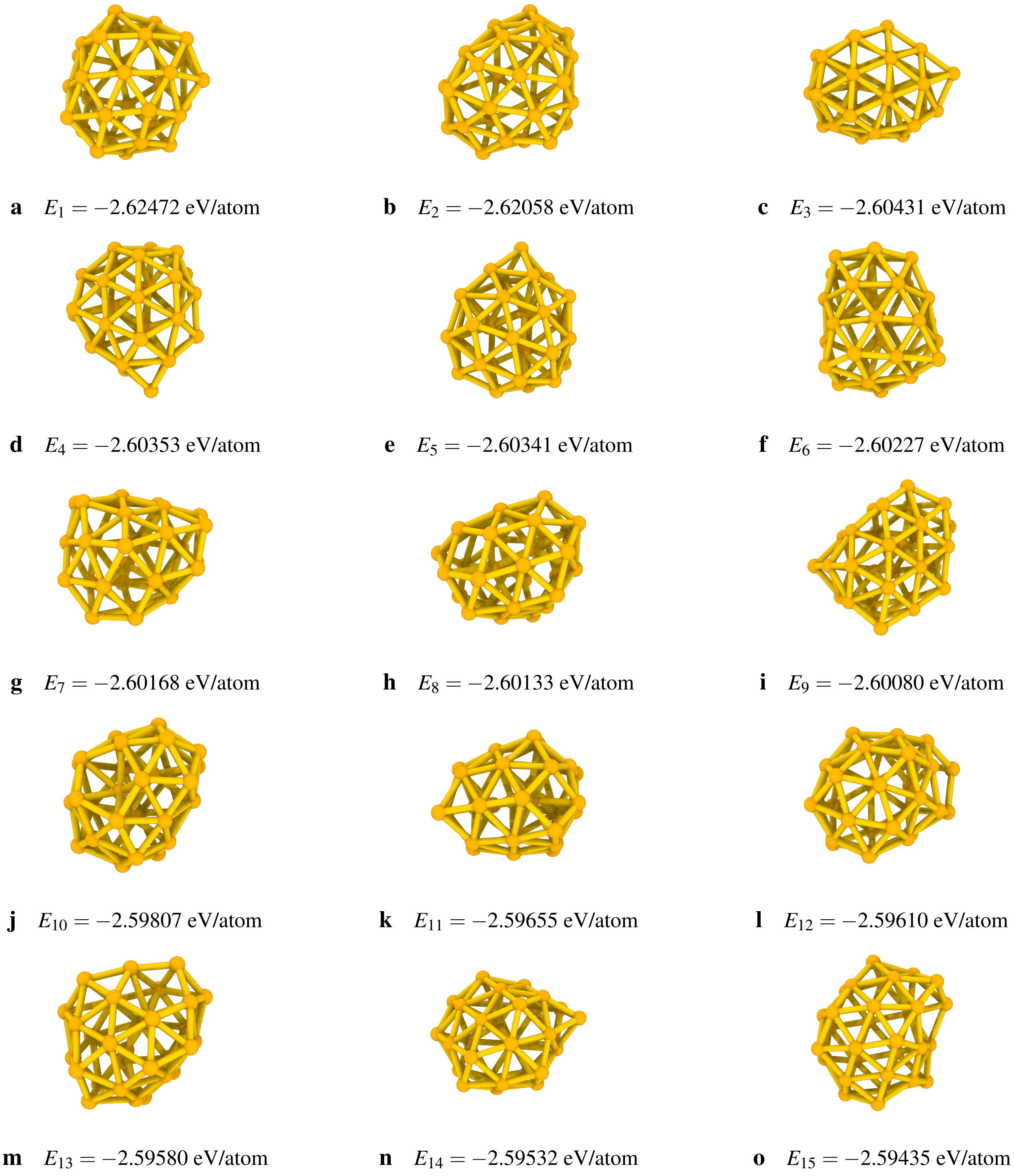

Figure S4 – Top 15 OMAT$_m$ Au$_{31}$ clusters. Shown are visualizations of OMAT$_m$ with $m \in \{1,\ldots,15\}$. These are the 15 most stable Au$_{31}$ clusters found by the adaptive diffusion evolution. Also given are the energies per atom E_m for every OMAT$_m$ structure.

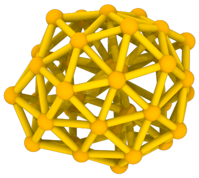

a $E_1 = -2.66799$ eV/atom

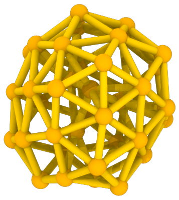

b $E_2 = -2.66681$ eV/atom

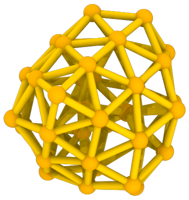

c $E_3 = -2.66406$ eV/atom

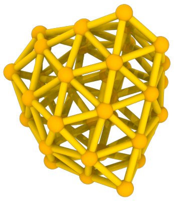

d $E_4 = -2.66028$ eV/atom

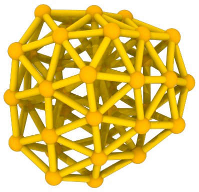

e $E_5 = -2.65760$ eV/atom

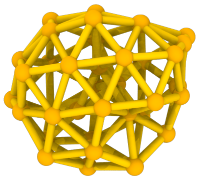

f $E_6 = -2.65747$ eV/atom

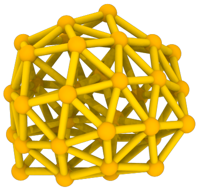

g $E_7 = -2.65674$ eV/atom

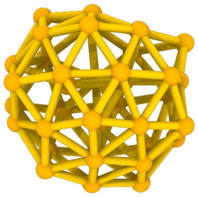

h $E_8 = -2.65558$ eV/atom

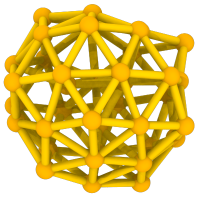

i $E_9 = -2.65558$ eV/atom

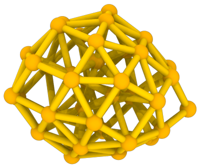

j $E_{10} = -2.65520$ eV/atom

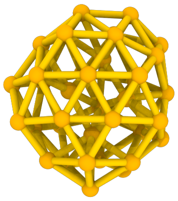

k $E_{11} = -2.65509$ eV/atom

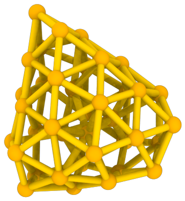

l $E_{12} = -2.65503$ eV/atom

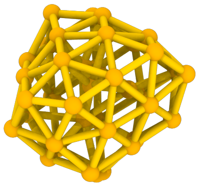

m $E_{13} = -2.65417$ eV/atom

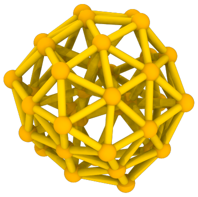

n $E_{14} = -2.65284$ eV/atom

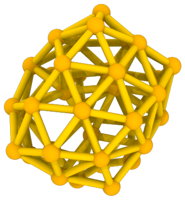

o $E_{15} = -2.65089$ eV/atom

Figure S5 – Top 15 OMAT$_m$ Au$_{38}$ clusters. Shown are visualizations of OMAT$_m$ with $m \in \{1, \ldots, 15\}$. These are the 15 most stable Au$_{38}$ clusters found by the adaptive diffusion evolution. Also given are the energies per atom E_m for every OMAT$_m$ structure.

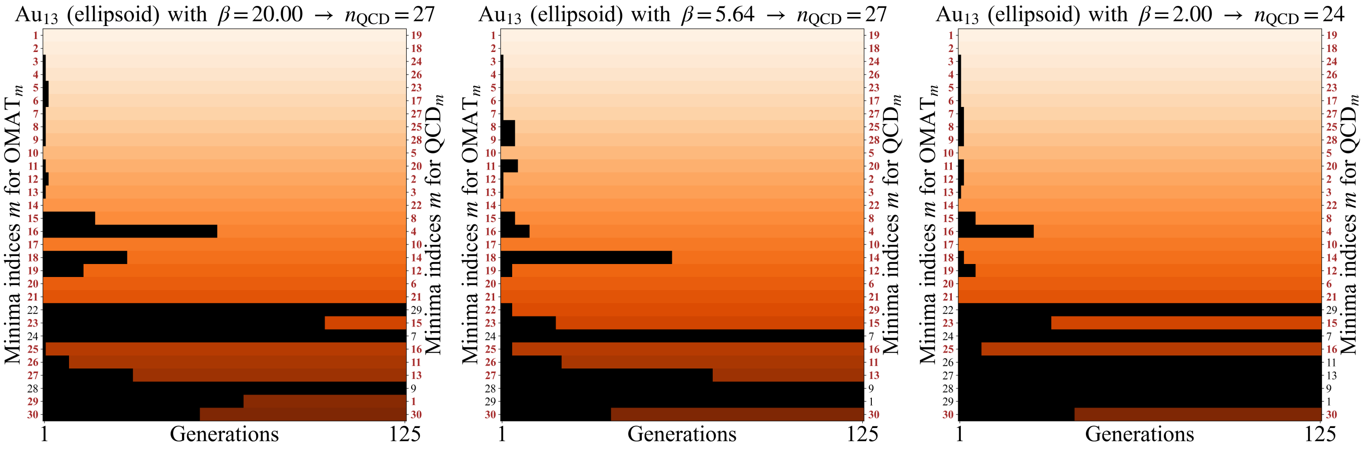

a Au_{13} (ell.), $\beta = 20.00$, $n_{\mathrm{QCD}} = 27$ **b** Au_{13} (ell.), $\beta = 5.64$, $n_{\mathrm{QCD}} = 27$ **c** Au_{13} (ell.), $\beta = 2.00$, $n_{\mathrm{QCD}} = 24$

Figure S6 – Comparison between OMAT-relaxed QCD minima and the results of an adaptive diffusion evolution. Shown are the indices of Au_{13} QCD cluster minima which have energies equivalent to structures found by an adaptive diffusion evolution (up to a difference of $\Delta E_{\mathrm{OMAT}} = 10^{-5}$ eV) if relaxed by the OMAT foundation model. The plots show in which generation these structures were found first. The colors of the rows indicate the stability of the minima, lighter colors being more stable. Structures that are not found are kept black during the whole evolution. The left axes depict the minima indices m as ordered by the OMAT foundation model, the right axes as ordered by the QCD database, in which colored indices identify found structures. Also given are the total number n_{QCD} of these configurations.

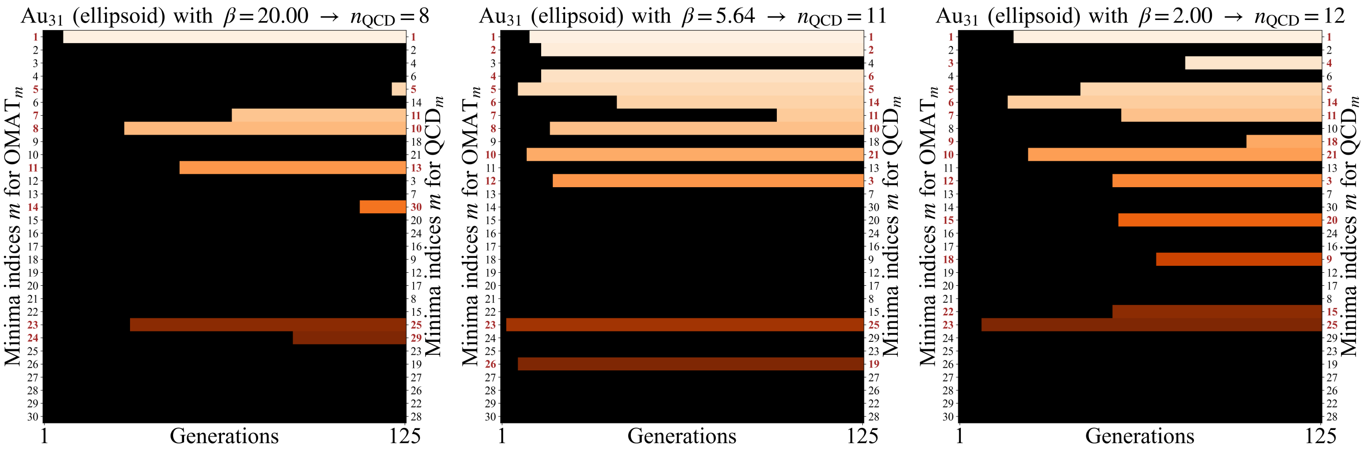

a Au$_{31}$ (ell.), $\beta = 20.00$, $n_{QCD} = 8$ **b** Au$_{31}$ (ell.), $\beta = 5.64$, $n_{QCD} = 11$ **c** Au$_{31}$ (ell.), $\beta = 2.00$, $n_{QCD} = 12$

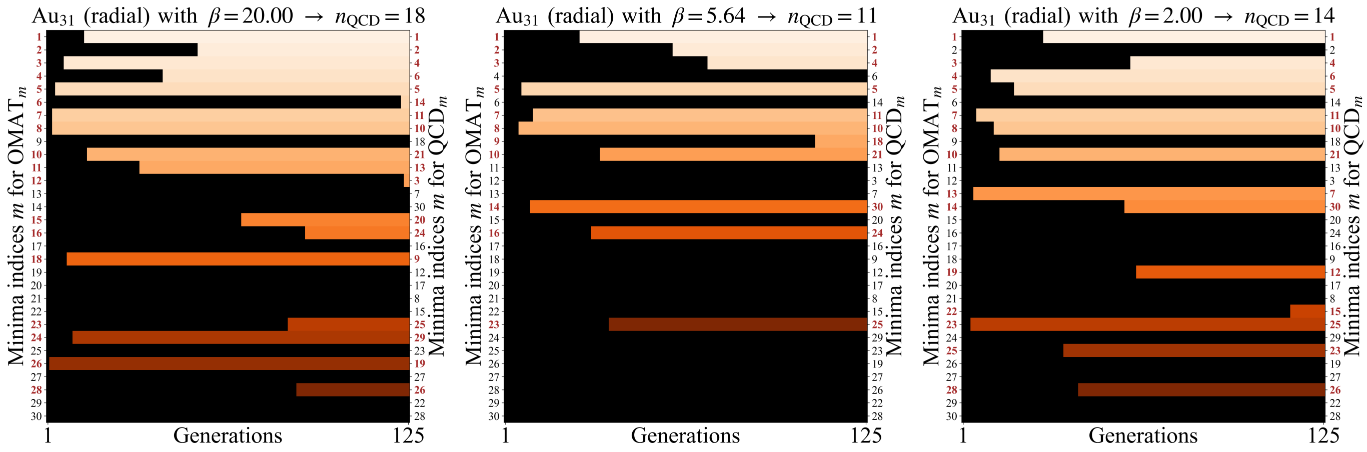

d Au$_{31}$ (rad.), $\beta = 20.00$, $n_{QCD} = 18$ **e** Au$_{31}$ (rad.), $\beta = 5.64$, $n_{QCD} = 11$ **f** Au$_{31}$ (rad.), $\beta = 2.00$, $n_{QCD} = 14$

Figure S7 – Comparison between OMAT-relaxed QCD minima and the structures found by an adaptive diffusion evolution. Shown are the indices of Au$_{31}$ QCD cluster minima which have energies equivalent to structures found by an adaptive diffusion evolution. (up to a difference of $\Delta E_{OMAT} = 10^{-5}$ eV) if relaxed by the OMAT foundation model. The plots show in which generation these structures were found first. The colors of the rows indicate the stability of the minima, lighter colors being more stable. Structures that are not found are kept black during the whole evolution. The left axes depict the minima indices m as ordered by the OMAT foundation model, the right axes as ordered by the QCD database, in which colored indices identify found structures. Also given are the total number n_{QCD} of these configurations.

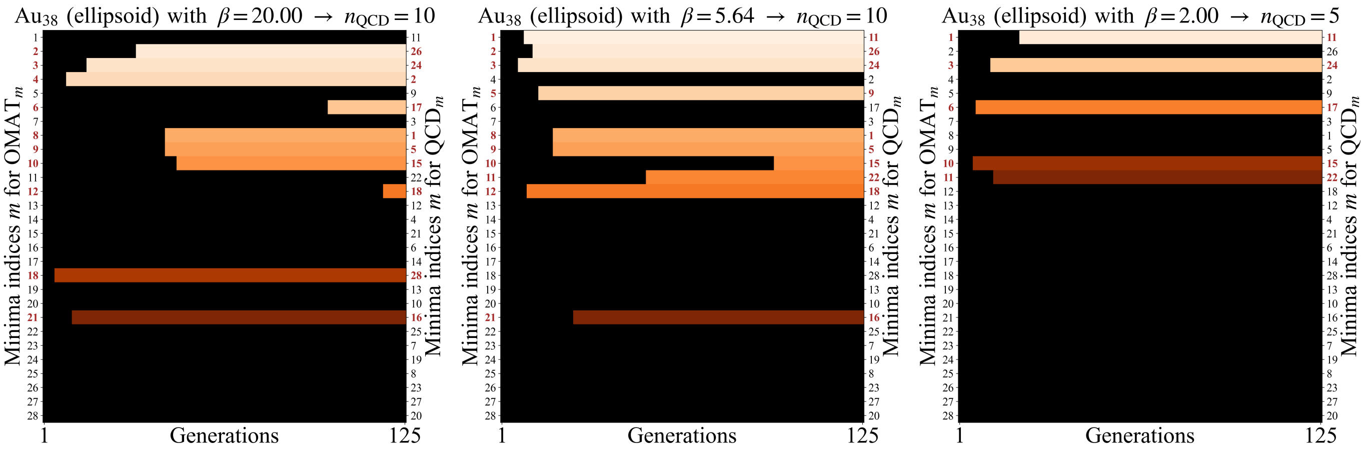

a Au_{38} (ell.), $\beta = 20.00$, $n_{QCD} = 10$ **b** Au_{38} (ell.), $\beta = 5.64$, $n_{QCD} = 10$ **c** Au_{38} (ell.), $\beta = 2.00$, $n_{QCD} = 5$

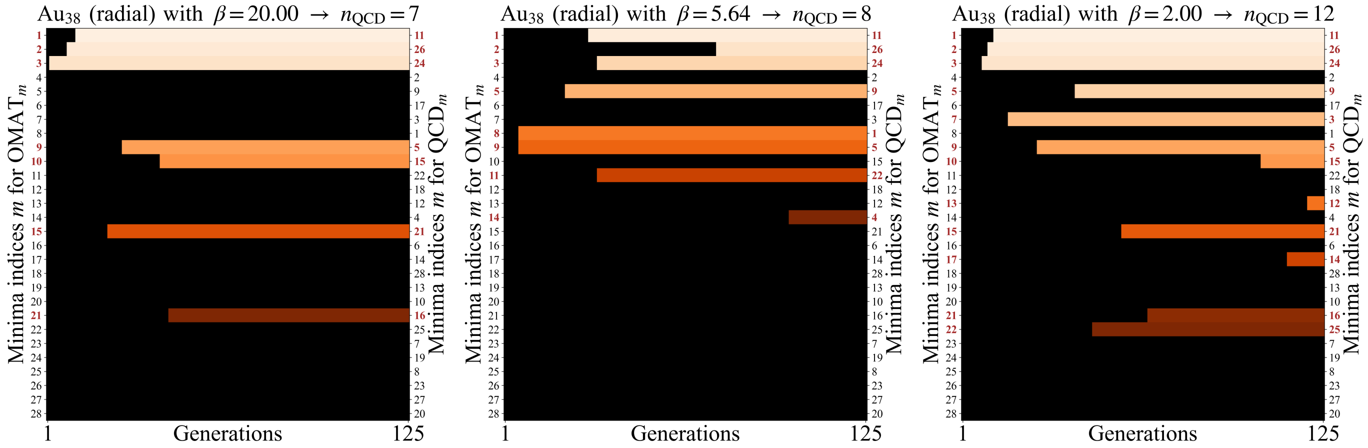

d Au_{38} (rad.), $\beta = 20.00$, $n_{QCD} = 7$ **e** Au_{38} (rad.), $\beta = 5.64$, $n_{QCD} = 8$ **f** Au_{38} (rad.), $\beta = 2.00$, $n_{QCD} = 12$

Figure S8 – Comparison between OMAT-relaxed QCD minima and an adaptive diffusion evolution. Shown are the indices of Au_{38} QCD cluster minima which have energies equivalent to structures found by an adaptive diffusion evolution. (up to a difference of $\Delta E_{OMAT} = 10^{-5}$ eV) if relaxed by the OMAT foundation model. The plots show in which generation these structures were found first. The colors of the rows indicate the stability of the minima, lighter colors being more stable. Structures that are not found are kept black during the whole evolution. The left axes depict the minima indices m as ordered by the OMAT foundation model, the right axes as ordered by the QCD database, in which colored indices identify found structures. Also given are the total number n_{QCD} of these configurations.
